# Evaluation of selected microbial and physicochemical parameters of fresh tomato juice after cold atmospheric pressure plasma treatment during refrigerated storage

**DOI:** 10.1038/s41598-019-44946-1

**Published:** 2019-06-10

**Authors:** Agnieszka Starek, Joanna Pawłat, Barbara Chudzik, Michał Kwiatkowski, Piotr Terebun, Agnieszka Sagan, Dariusz Andrejko

**Affiliations:** 1 Department of Biological Bases of Food and Feed Technologies of University of Life Sciences in Lublin, Głęboka 28, 20-612 Lublin, Poland; 2 Institiute of Electrical Engineering and Electrotechnologies of Lublin, University of Technology, Nadbystrzycka 38a, 20-618 Lublin, Poland; 3 Department of Cell Biology, Institute of Biology and Biochemistry, Maria Curie-Skłodowska University, Akademicka 19, 20-033 Lublin, Poland

**Keywords:** Antimicrobials, Nutritional supplements, Fluids

## Abstract

The Cold Atmospheric pressure Plasma (CAP) technology is an emerging technology used for conditioning and microbiological decontamination of biomaterials including food. A novel tool for inactivation of juice background spoilage microorganisms, as well as high count of inoculated yeast while maintaining physicochemical properties in tomato juice - CAP technology was utilized in this study. Dry matter content and pH were not significantly influenced by CAP generated in GlidArc reactor. Small increase of lycopene, and slight loss of vitamin C content were observed.

## Introduction

Cold Atmospheric pressure Plasma (CAP) produced as a result of electrical discharges is a unique state of matter with unique properties of ionized gas. Non-equilibrium atmospheric pressure plasma discharges can generate many biochemically active compounds in surrounding gaseous environments, and as a result of plasma interaction with treated materials^1–3^ including liquid media. The compounds produced under the influence of CAP in juices have not been extensively studied so far, but research carried out in liquids including the complete culture media have demonstrated the generation of many biochemically active ingredients like charged particles (ions and electrons), free radicals and reactive oxygen and nitrogen species (RONS), including atomic nitrogen (N), nitric oxide (NO), peroxynitrite (ONOO^−^) and derived species, atomic oxygen (O), hydroxyl radical (·OH), superoxide (O_2_^−^) and hydrogen peroxide (H_2_O_2_)^4–12^. It is well-proven that the mentioned components have bactericidal and fungicidal effects; therefore the possibility of sterilization of food, especially fruit and vegetable juices, with CAP treatment is an issue of great interest. The use of CAP as a sterilization technique has many advantages, including low costs, avoidance of chemicals and undesirable changes in products associated with heat treatment.

Attempts to use CAP for surface sanitization of in-package fruit, vegetables, meat, and other food products such cereal grains have shown promising results, indicating effective eradication of microorganisms, prolonging the shelf life of the products, reducing spoilage losses and improving nutritional, functional and sensory properties of food products^13–17^. Microbiological decontamination of liquid products such as juices, milk and dairy products until recently was considered to be ineffective due to the rapid deactivation of plasma components by organic molecules contained in solutions. Research has shown, however, that active molecules induced by CAP in aqueous solutions can effectively inactivate the pathogenic and spoilage microorganisms without the need for direct exposure to the plasma. The influence of plasma on the functional and nutritional properties of food products remains debatable. The impact of CAP on food products has been studied carefully in milk and milk products such as chocolate milk drink and whey beverage^13,18–20^. The authors report that mild CAP conditions increase the bioactive and volatile compounds, as well as antioxidant capacity, while maintaining a satisfactory lipid profile. However, a more drastic CAP treatment leads to loss of vitamins and volatile compound and unfavorable changes in the lipid profile. Depending on the intensity of the CAP treatment, the denaturation and formation of protein aggregates was also observed, resulting in milk beverages with different consistencies.

Previous studies have repeatedly proven the antibacterial and antifungal activity of CAP, ut in the literature there are few reports on its effectiveness in the microbial decontamination of fruit or vegetables juices. Available studies were conducted using pasteurized juices inoculated with a specific microorganism. Reduction *of Escherichia coli* counts was tested in orange, tomato, and apple juices and in sour cherry nectar^21,22^, *Salmonella enterica* in orange juice^23^, *Citrobacter freundii* in apple juice^24^ and *Sccharomyces cerevisiae* in white grape juice^25^. The CAP effectiveness in the elimination of naturally occurring background microflora in unpasteurised juice, responsible for its spoilage, has not been studied so far. Elimination of yeasts responsible for spoilage of food products such as juice is an issue of great interest^26,27^.

Examination of fruit and vegetable juices treated with CAP for microbiological decontamination indicates that the organoleptic and physicochemical properties, such as color, pH, acidity, electrical conductivity, Brix, do not change^13,25,28,29^. Ma and Lan^28^ showed that the content of volatile components, analyzed by gas chromatography and mass spectrometry, did not differ significantly from the composition of fresh tomato juice, in contrast to the thermally treated juice. There was reported a slight increase in enzymatic darkening of white grape juice and apple juice, which did not significantly affect its consumer value^22,30^. Many authors have shown that optimized CAP treatment conditions can improve the nutritional and taste values of fruit and vegetables juices. Fernandes *et al*.^30^ showed that under the influence of nitrogen glow plasma in acerola juice, the content of vitamin A and carotenoids increased, while having a very small effect on the content of vitamin C and antioxidant capacity. At the same time, the authors noted a reduction of phenolics content by 30%. Treatment of cashew apple juice with nitrogen plasma resulted in increased content of vitamin C, flavonoids, polyphenols, and increased antioxidant capacity^31^. The increase in total flavonols and total phenolic content was also reported in other types of juices^15,22^. On the other hand, spectrophotometric analysis of white grape juice showed that 4-minute CAP processing caused a decrease in total phenolics and flavonoids, DPPH free radical scavenging and antioxidant capacity, comparable to samples of thermally treated juice^25^. Xu *et al*.^23^ reported that CAP treatment of orange juice causes a loss of 22% in vitamin C, compared with a 50% reduction in thermally treated juice.

Inconsistent literature data on the impact of CAP treatment on the organoleptic and nutritional value of fruit and vegetable juices is caused by various parameters of plasma processing, such as the type of reactor, time of CAP treatment, sample volume, distance from the nozzle, type of gas used, etc. The authors point to the need to optimize process, as overexposure leads to the loss of valuable juice ingredients^21,31^. The intensity of plasma treatment should be optimized in order to effectively inactivate pathogenic and spoilage microorganisms and, on the other hand, to keep as many unchanged chemical components as possible. For this reason, studies on the effectiveness of CAP in eliminating microorganisms should be supplemented each time with the biochemical studies of the product properties.

The aim of the present work was to examine the impact of CAP treatment on the quality and physicochemical properties of the fresh tomato juice and its effectiveness in inactivation of the background microflora and high counts of the spoilage yeasts. The CAP usefulness in prolonging the shelf-life of fresh tomato juice with minimized losses of valuable nutrients was also evaluated.

## Materials and Methods

### Preparation of tomato juice

Fresh tomatoes (*Lycopersicon Esculentum cv*. *Apis F1*) were purchased from a local fruit market, Lublin, Poland. The tomatoes were washed with running tap water, dried with paper towels, and then cut into four pieces with a stainless steel knife. Tomato juice was extracted using a slow juice extractor SSJ 4043WH, Sencor, Říčany, Czech Republic (juice and seeds were removed automatically).

The juice was divided into five different parts as a control sample and working samples for the atmospheric plasma treatment. Samples of the juice were used for microbiological and physicochemical analysis.

Pasteurized tomato juice (not from concentrate) was purchased in a bio-market. It was divided into five different parts as a control sample and working samples for the atmospheric plasma treatment. Samples of the pasteurized juice were used for microbiological analysis.

### Plasma treatment system

**Figure 1:**
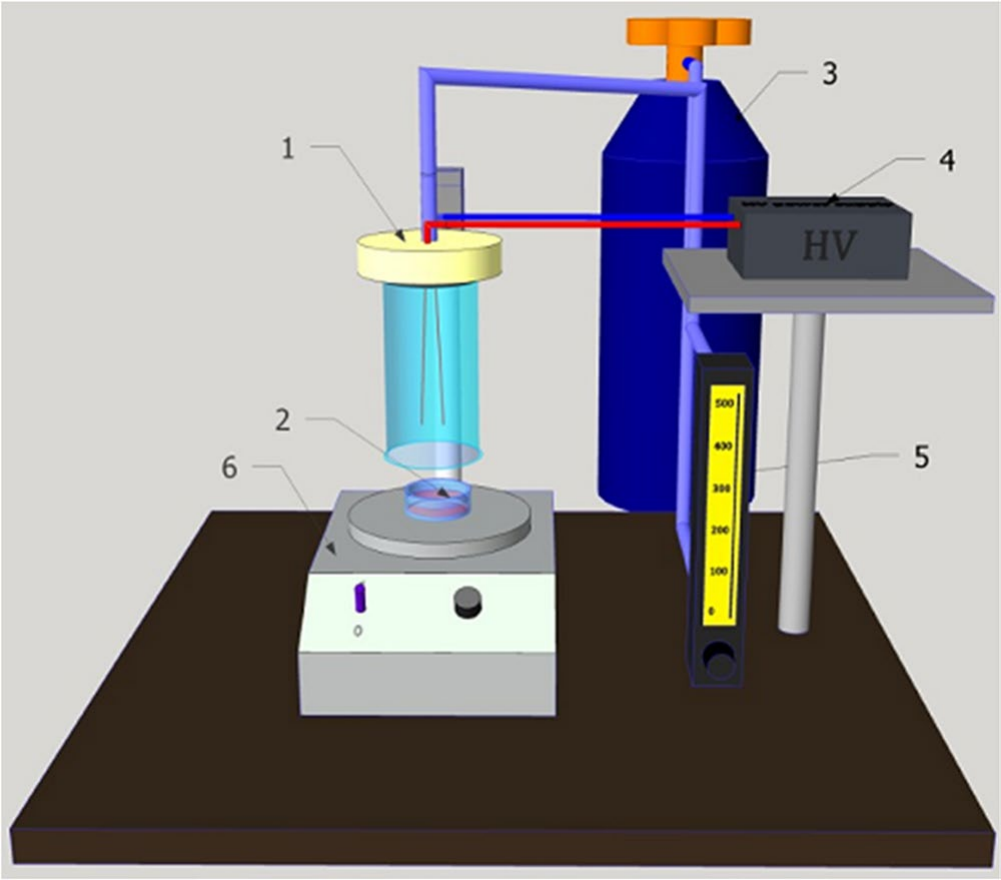
Schematic diagram of the atmospheric plasma treatment system (1- GAD, 2- sample, 3- gas supply, 4- power supply, 5- flowmeter, 6- magnetic stirrer).

**Figure 2:**
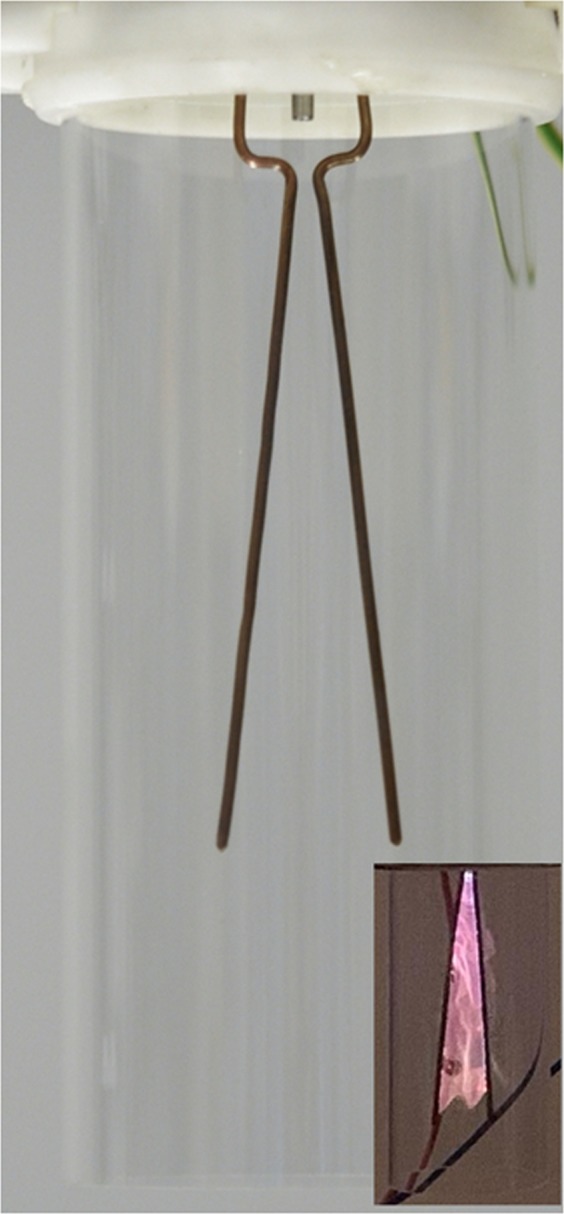
Photo of GAD reactor.

AC powered GlidArc reactor (GAD) operated at the atmospheric pressure with frequency of the discharge of 50 Hz, applied voltage amounting to 3.8 kV and mean power ranging 40 W. The length of 2 diverging profiled copper rod electrodes was 10 cm. Inter-electrode distance (closes point between top of electrodes) was set at minimum of 3 mm^32–34^. The flow rate of the nitrogen gas (purity 6.0, Linde Gas Poland) was adjusted by gas flow controller to 440 L/h. The experimental set-up and photo of reactor are presented in Figs 1 and 2. Air was used as substrate gas.

### Microbiological analysis

#### Preparation of tomato juice samples and plasma treatment

In the first variant of the experiment, un-pasteurized tomato juice freshly made in a juice extractor was aliquoted into 5 ml samples and poured into a sterile glass container (Ø 2,7 mm). It was placed on a magnetic stirrer on an ice bath and treated with non-thermal atmospheric pressure plasma for 30, 60, 120, and 300 seconds. The nozzle was set 1 cm from the surface of the juice and was surrounded by a cylindrical glass cover to prevent dispersion of active plasma molecules in the environment. Temperature, measured using uninsulated K-type thermocouple with electronic temperature compensation multimeter was below 30 °C for all experimental settings.

All operations were done in aseptic conditions. The control consisted of plasma-untreated freshly made un-pasteurised tomato juice samples.

In the second experimental variant, the pasteurized tomato juice purchased from a local store was inoculated with *Candida albicans* cells (strain NCPF 3153, National Collection of Pathogenic Fungi) or *Saccharomyces cerevisiae* baker’s yeast. To obtain yeast cultures in the logarithmic growth phase, the cells stored at −70 °C in a cryoprotectant were inoculated into liquid Yeast extract Peptone Dextrose (YPD) medium (10 g/L yeast extract, 20 g/L bacteriological peptone, 20 g/L glucose, 15 g/L agar) and cultured in 2.0 ml Eppendorf round-bottom vials at 35 °C, with shaking for 24 h. The density of the early stationary phase culture obtained in this way was determined using the Bürker hemocytometer chamber and a bright-field microscope. After determining the cell density, the culture suspension was used for inoculation of the pasteurized tomato juice samples in a calculated volume to obtain the final density of 5 ×10^4^ cells/ml. Immediately after the inoculation, the tomato juice was aliquoted into 5 ml samples, poured into a sterile glass container, and treated with cold atmospheric plasma according to the procedure described above. The control consisted of plasma-untreated pasteurized tomato juice samples. After the plasma treatment, all juice samples were poured into sterile Falcon tubes and kept in a refrigerator at 4 °C.

### Microorganisms counting

The un-pasteurized tomato juice samples, control and CAP-treated, were analyzed for background microflora, including the total aerobic mesophilic viable count and the total yeast and mold count. Samples of pasteurized tomato juice inoculated with *C*. *albicans* or *S*. *cerevisiae* at the initial density of 5 × 10^4^ cells/ml (4.7 log_10_ cells/ml) were analyzed for the number of colony forming units of the inoculated yeast. All microbiological cultures were made on days 1, 2, 3, and 5 of storage in a refrigerator at 4 °C after the treatment of the juice samples with CAP. Ten-fold serial dilutions of juice samples were prepared aseptically in a Laminar flow hood by transferring 1 ml of each juice sample into 9 ml of sterile phosphate buffered saline. 100 µl aliquots from each dilution were spread using a sterile L-a shape spreader on the surface of nutrient agar medium and incubated for 72 h at 30 °C for total aerobic viable counts or on the surface of Sabouraud agar with penicillin and streptomycin and incubated for 5 days at 25 °C for total yeast and mold counts. To count the inoculated *C*. *albicans* and *S*. *cerevisiae*, 100 µl aliquots from serial dilutions were spread on the surface of Sabouraud dextrose agar and the plates were incubated for 48 h at 35 °C. After an appropriate incubation time, the colonies were counted and, taking into account dilution, colony forming units per milliliter (CFU/ml) were determined.

### Physicochemical analysis

#### Determination of pH

The pH of the tomato juice samples was measured using a digital pH meter (780 pH Meter, Metrohm, Herisau, Switzerland) previouslycalibrated with commercial buffer solutions of pH 7.0 and 4.0. 10 mL of the sample was put into a beaker and stirred continuously using a magnetic stirrer, pH was measured at 25 ± 0.5 °C.

#### Determination of dry weight

Thermal drying method using a Dryer type SLN15 STD, Wodzisław Śląski, Poland was employed to measure the dry mass of the samples. Thermal drying at the temperature of 105 °C in normal pressure conditions^35^ is applied to remove water present in the product effecting in decrease of mass.

#### Determination of total carotenoids and lycopene

Total carotenoid and lycopene content were determined by spectrophotometry using a spectrophotometer Thermo Scientific UV-Vis Helios Omega 3, Waltham, Massachusetts, USA. The absorbance of the organic phase for the total carotenoid content was measured in triplicate at 470 nm versus a blank of hexane. The absorbance for lycopene in the extracts was measured at 503 nm using hexane as a blank^36^.

#### Determination of ascorbic acid

The content of vitamin C (L-ascorbic acid) was determined using 2,6-dichlorophenolindophenol (Tillmans dye) in accordance with Hallmann^35^. The sample was extracted in 2% oxalic acid. The solution was filtered. The filtrate was collected and then titrated with the Tillmans dye until a permanent pink color was reached.

### Statistical analysis

For microorganisms counting the CAP-treatment experiment was repeated two times, and the number of colony forming units (CFU/ml) was checked in triplicate for each experimental sample. Data obtained from microbial counts were tabulated using Microsoft Excel (MS Excel 2010, Microsoft Corporation). Results are expressed as mean values (n = 6) with standard deviation (SD) of log_10_ of the colony forming units per milliliter (log_10_ CFU/ml). For physicochemical studies experiments were carried out in triplicate and the data are presented as a mean value ± SD. Statistical analysis of the data was performed with Statistica software^37^ using analysis of variance for factorial designs (ANOVA). The significance of differences was tested using a Tukey LSD test (P ≤ 0.05).

## Results and Discussion

### Microbiological analysis

**Figure 3:**
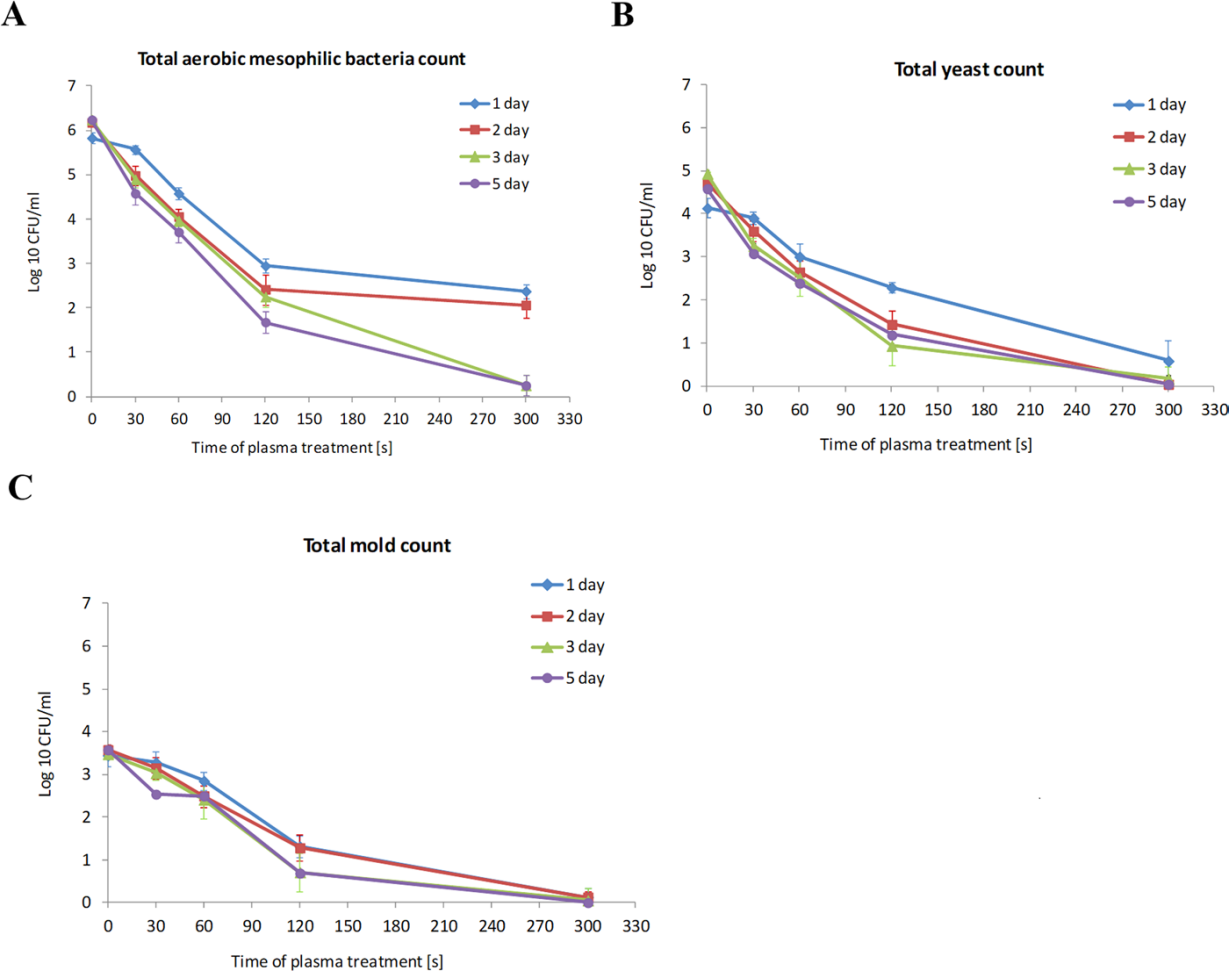
Background microbial load (log_10_ CFU.ml) of un-pasteurized tomato juice treated with non-thermal atmospheric pressure plasma (air) for 0, 30, 60, 120, and 300 s and stored at +4 °C for 1–5 days. (**A**) Total aerobic mesophilic bacteria count; (**B**) total yeast count; (**C**) Total mold count. Mean values with SD from two independent experiments made in triplicate are presented.

**Figure 4:**
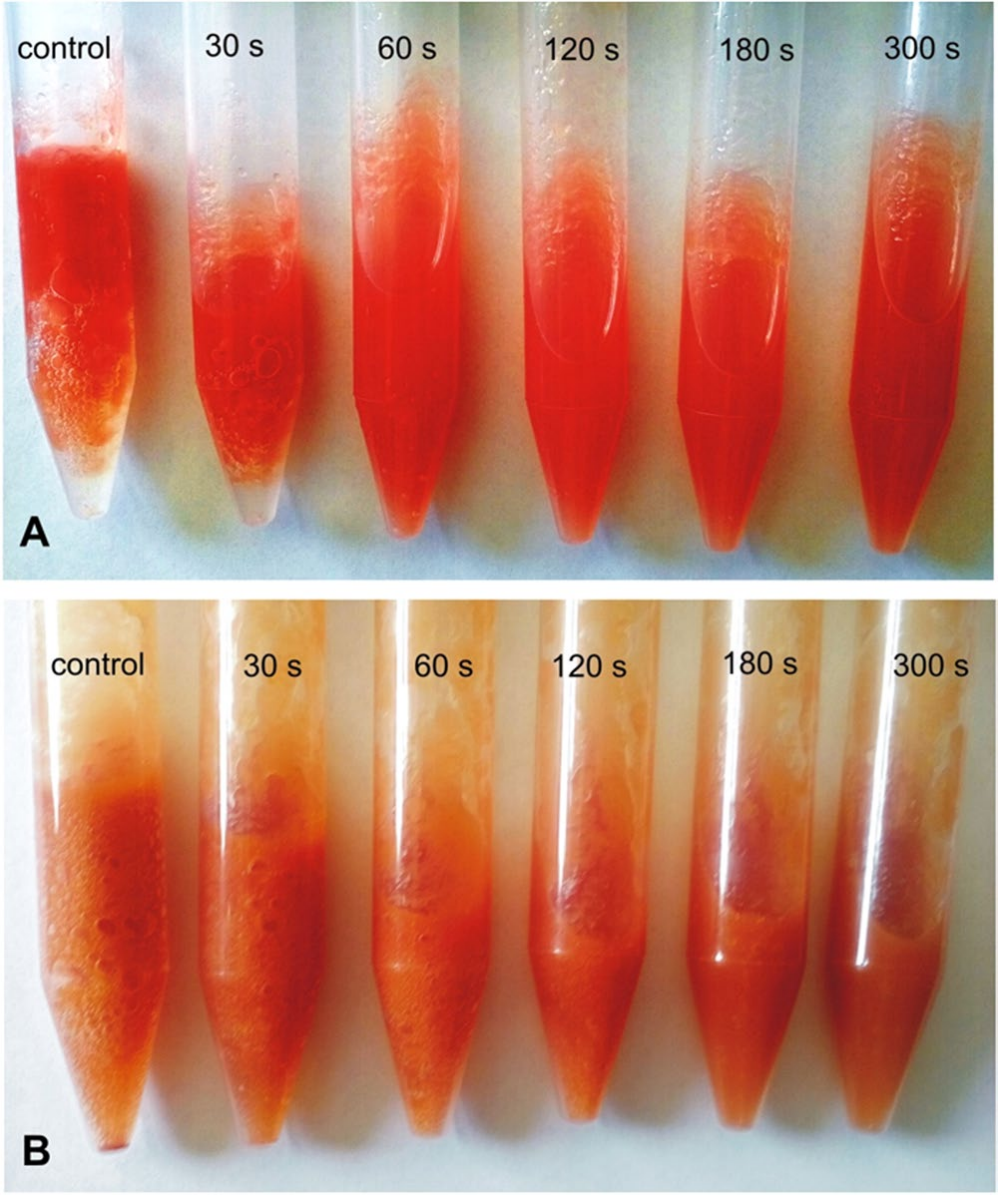
Photographs of tomato juice samples exposed to cold atmospheric plasma for the time indicated in the photograph. (**A**) Un-pasteurized juice photographed on the on the fifth day after plasma treatment; gas bubbles and juice stratification (visible in the samples exposed for 0 and 30 s) indicate the spoilage of the juice. (**B**) Pasteurized juice inoculated with *S*. *cerevisiae* (5 × 10^4^ cells/ml) photographed on the first day after plasma treatment; the CO_2_ bubbles (visible in the samples exposed for 0, 30 and 60 s) indicate the fermentation activity of *S*. *cerevisiae* cells.

The total aerobic viable count is a measure of the microbial quality of a vegetable juice. The regulation applicable in Poland defines the maximum number of aerobic mesophilic microorganisms at the level of 10^3^–10^4^ CFU/ml only in pasteurized fruit and vegetable juices^38^. The regulation of the European Commission (EC) specifies only the limit of bacteria from the group of *E*. *coli* in un-pasteurized fruit and vegetable juices^39^. There is limited information regarding the permissible number of total aerobic microflora in freshly squeezed, un-pasteurized tomato juice. The data available in the literature on the total number of microorganisms in fresh fruit and vegetable juices differ greatly (from 2 to 7 log10 CFU/ml) depending on the method of juice production, the type of fruit and vegetable cultivation, variety, and storage conditions^40–43^. Generally, it is believed that the presence of microorganisms in a high number (>4 log10 CFU/ml) is responsible for juice spoilage. The present study investigates the microbiological quality of un-pasteurized tomato juice obtained at home using a juicer and the effectiveness of cold atmospheric plasma treatment in eradication of the microbial load of this juice. In the control samples of unpasteurized tomato juice inoculated on the nonselective nutrient agar, a very large number of aerobic mesophilic microflora colonies were found. The mean total aerobic viable count of 5.83 ± 0.12 log_10_ CFU/ml after 1 day of storage in a refrigerator at +4 °C increased to 6.24 ± 0.04 log_10_ CFU/ml after 3 days of storage and remained at a similar level after 5 days of storage. In the cultures on the selective Sabouraud agar medium with penicillin and streptomycin, a large number of yeasts colonies (4.14 ± 0.22 log_10_ and 4.94 ± 0.02 CFU/ml after 1 and 3 days of storage, respectively) and molds (3.45 ± 0.26 log_10_ and 3.48 ± 0.29 CFU/ml after 1 and 3 days of storage, respectively) were detected as well. The organoleptic evaluation of non-pasteurized tomato juice showed a change in the color into brownish-red and a smell of spoiled juice was detectable after 5 days of storage at +4 °C. The use of cold atmospheric plasma immediately after squeezing the juice proved to be very effective in eradication of background microflora. In our experiment, the basic determinant of the effectiveness of the biological decontamination of the tomato juice by the cold atmospheric plasma was the time of exposure. In the cultures made on the first day after the treatment with plasma, statistically significant reduction (*p* < 0.05) in the number of CFU/ml was obtained after only 30 s of the plasma treatment for the total viable count and after 60 s for the yeast and mold count. However, the reduction of the number of microorganisms by more than 3 log, which is satisfactory from the point of view of decontamination, was obtained after 5 minutes of plasma treatment: 3.45-log CFU/ml reduction for the total aerobic mesophilic bacteria colonies (Fig. 3A), 3.55-log CFU/ml reduction for yeast (Fig. 3B), and 3.32-log CFU/ml reduction for molds (Fig. 3C). In the case of yeast and mold, almost complete eradication of the colony forming units was obtained after 5 minutes of plasma treatment. There was also an advantageous effect of reduction in the total number of microorganisms on the following days of storing the plasma-treated juice at 4 °C, in contrast to the control juice (Fig. 3A–C). After 2, 3, and 5 days of juice storage in refrigeration conditions, a satisfactory reduction in the number of microorganisms by more than 3 log was noted in samples treated with plasma for only 2 minutes (3.76-log, 3.99-log, and 4.56-log CFU/ml reduction after 2, 3, and 5 days of storage, respectively, for the total viable count). In the juice samples treated with plasma for 5 minutes, almost complete eradication of the total viable count was recorded after 3 and 5 days of storage, and the number of colonies was reduced by 5.99 and 5.94 log CFU/ml (Fig. 3A). Very similar results were obtained for the yeast and mold count (Fig. 3A,B). After 5 days of storing the plasma-treated juice samples for 2, 3 and 5 minutes, the juice still retained an intense red color and a fresh smell, which indicates effective inactivation of the background microflora and the absence of the spoilage phenomenon (Fig. 4A). Results of organoleptic evaluation of non-pasteurized tomato juice stored at +4 °C, depending on the time of plasma treatment were summarized in Table 1. The phenomenon of reducing the total number of microbial colony forming units during storage under refrigeration of the plasma-treated tomato juice samples is probably caused by the prolonged exposure of microbial cells to the active species generated in the juice under the influence of plasma. The phenomenon of progressive inactivation of the microorganisms during the following hours of storage of apple juice treated with cold atmospheric plasma has been described for *Citrobacter freundii* by Surowsky *et al*.^24^. The authors of this work observed after immediate plating after 480 s of plasma exposure small inactivation rates ranging 1 log cycle. In comparison, a 4.4-log cycle reduction was observed after 3-h storage time. Extension of the storage time to 24 h resulted in 5.1 log cycles reduction. The progressive inactivation of microorganisms during storage of the plasma-exposed juice may be caused by the generation of active antimicrobial ingredients, including hydrogen peroxide (H_2_O_2_), hydroperoxy radicals (OOH•), peroxynitrite (ONOO−) and derived species, which have extended lifetimes, compared to OH and O radicals, and remain still active in the plasma-treated juice^4,5,24,44^. In addition, tomato juice has low content of sugars that are necessary for cells to trigger effective defense mechanisms against oxidative stress induced by the active plasma components. The lack of a sufficient energy source, e.g. sugars, can cause accelerated death of microbial cells impaired by plasma.

**Table 1:** Organoleptic evaluation of non-pasteurized tomato juice stored at +4 °C, depending on the time of plasma treatment.

Time of plasma treatment [s]	Analysis day	Smell	Color
0 (control)	1	fresh	intense red
	2	fresh	intense red
	3	spoiling	brownish red
	5	clearly spoiled	brownish red
30	1	fresh	intense red
	2	fresh	intense red
	3	spoiling	brownish red
	5	clearly spoiled	brownish red
60	1	fresh	intense red
	2	fresh	intense red
	3	spoiling	brownish red
	5	spoiling	brownish red
120	1	fresh	intense red
	2	fresh	intense red
	3	fresh	intense red
	5	fresh	intense red
300	1	fresh	intense red
	2	fresh	intense red
	3	fresh	intense red
	5	fresh	intense red

In the second part of the experiment, the pasteurized tomato juice was inoculated with a suspension of *C*. *albicans* or *S*. *cerevisiae* yeast cells with a known density and then treated with the plasma in conditions identical to those described earlier for the unpasteurized juice. The aim of this experiment was to check the effectiveness of the sterilizing effect of the plasma against the high density of yeast cells. The choice of the yeast was determined by the fact that they occur naturally on fruits and vegetables and they are largely responsible for spoiling and fermenting juices. Our results have shown that the cold atmospheric plasma treatment is effective in eradication of high-density yeasts in tomato juice, but the time of the treatment needed for yeast inactivation depends on the species.

**Figure 5:**
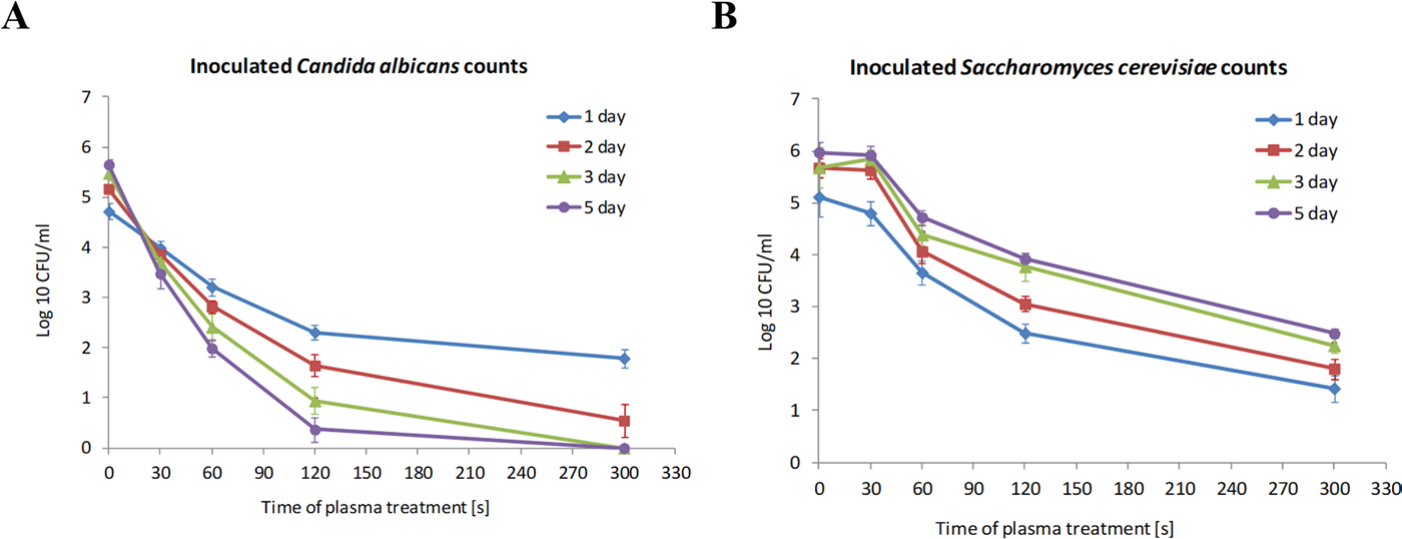
Total inoculated yeast counts (log_10_ CFU/ml); in pasteurized tomato juice treated with non-thermal atmospheric pressure plasma for 0, 30, 60, 120, and 300 s stored at +4 °C for 1–5 days. (**A**) *Candida albicans*; (**B**). *Sachcaromyces cerevisiae* Mean values with SD from two independent experiments made in triplicate are presented.

In the control pasteurized juice samples inoculated with *C*. *albicans* at the initial density of 5 ×10^4^ cells/ml (4.70 log_10_ cells/ml) after 5 days of storage at 4 °C, the number of CFU/ml increased to 5.65 log_10_ CFU/ml (Fig. 5A). Microbial cultures on Sabouraud dextrose agar performed on the first day after the plasma treatment showed a statistically significant reduction in the number of CFU/ml compared to the control after only a 30-s treatment time. However, a satisfactory 2.93-log_10_ CFU/ml reduction was observed after 5 min of the plasma treatment. Importantly, as in the case of the naturally occurring microflora described earlier, the number of *C*. *albicans* CFU/ml decreased in the plasma-treated juice samples during storage in refrigeration conditions. Reduction in the number of colonies by more than 3 log_10_ was recorded after 2 days of storage in samples treated with plasma for 2 min (3.53-log_10_ CFU/ml reduction) and after 3 and 5 days of storage in samples treated with plasma for 1 min (3.07-log_10_ and 3.66- log_10_ CFU/ml reduction, respectively) (Fig. 5A). It should be noted that, after 3 and 5 days of storage in the refrigerator, there was almost complete eradication of the *C*. *albicans* colonies in the juice samples treated with plasma for 2 and 5 min.

In the juice inoculated with the yeast *S*. *cerevisiae*, the results were less satisfactory than in the case of background microflora and the yeast *C*. *albicans*. In the juice samples inoculated with *S*. *cerevisiae* and not treated with plasma, propagation of yeast cells was observed, the number of which increased by 1.28 log_10_ CFU/ml after 5 days of storage at 4 °C (Fig. 5B). A statistically significant reduction in the number of colonies was obtained in this experiment in juice samples treated with cold atmospheric plasma for 1 min, whereas the treatment for 30 s did not give this effect. The reduction in the number of colonies by more than 3 log_10_ was observed in samples treated for 5 minutes with plasma (3.69-log 10 CFU/ml reduction on the 1st day) (Fig. 5B). In the case of *S*. *cerevisiae*, there was no beneficial effect of reduction of the number of cells during storage of the plasma-treated juice. The number of *S*. *cerevisiae* colonies increased slightly during storage, but after 5 days in the samples treated with plasma for 5 minutes the number of CFU/ml declined by 3.49 log_10_, compared to control. There was a further beneficial effect of a significant decrease in the metabolic activity of *S*. *cerevisiae* cells in the samples treated with plasma for 2 min or longer, as shown by the production of CO_2_ bubbles in the anaerobic fermentation of this yeast (Fig. 4B). After 5-day storage of samples treated with plasma for 5 min, no significant increase in gas bubble formation was observed, as opposed to the control and samples treated with plasma for a shorter time. These results demonstrate the effectiveness of the cold atmospheric plasma treatment in inactivation of *S*. *cerevisiae* yeast cells.

The results of our experiments show that the effectiveness of a particular type of cold plasma depends primarily on the time of its application, but also on the specific sensitivity of the microorganism under study and the number of its cells. The experiment with microflora naturally occurring in tomato juice clearly shows that the cold atmospheric plasma is effective in elimination of microorganisms and its use can extend the shelf life of thermally untreated fresh tomato juices. When designing juice sterilization using plasma, however, the conditions should be selected very carefully, taking into account the type of plasma generator, the type of gas used, juice sample volume, stirring, and the time of treatment.

### Physicochemical analysis

pH is one of the crucial parameters of tomato juice because it influences the quality of juice during storage. There was no significant difference (P > 0.05) between the pH of the control samples (4.57) and tomato juice treated with atmospheric cold plasma for 30 (4.56) and 60 seconds (4.55) (Table 2). The increase in pH was to some extent higher for 120 and 300 seconds of plasma treatment. The pH values are in agreement with other researcher’s results. Prebiotic orange juice was treated with plasma and the pH values of the treated samples were statistically different from non-treated sample, which served here as control. The mean pH value obtained for the control was 4.43 and the pH values for treated orange juice samples ranged from 3.9 to 4.0. The change in pH is presumably an effect of the loss of water and metabolic changes in the tomato juice. On the other hand, the pH of ozone-treated samples was not statistically different from control sample, which was not treated by O_3_. Surprisingly, in spite of different ozone loads applied for processing of juice, pH was not affected^45^. In the case of grape juice, control sample pH was 3.38, while pH ranging 3.30–3.38 was observed after high voltage atmospheric cold plasma treatment^25^.

**Table 2:** Effect of atmospheric pressure plasma treatment (air) on pH, dry matter, total carotenoid, lycopene and ascorbic acid content in tomato juice.

Samples	pH	Dry matter (%)	Carotenoids (mg/100 g d.w)	Lycopene (mg/100 g d.w)	Ascorbic acid (mg/100 g d.w)
Control	4.57 ± 0.04a	3.69 ± 0.04b	131.5 ± 0.71a	118.5 ± 0.50a	274.00 ± 4.24ab
30 seconds treatment	4.56 ± 0.01a	3.44 ± 0.04c	135 ± 2.83ab	119.00 ± 1.00a	286.00 ± 4.24b
60 seconds treatment	4.55 ± 0.00a	3.55 ± 0.04ac	140 ± 1.41b	122.00 ± 1.00a	279.00 ± 2.83b
120 seconds treatment	4.71 ± 0.01b	3.59 ± 0.03ab	148.5 ± 2.12c	134.50 ± 0.50b	263.00 ± 2.83a
300 seconds treatment	4.68 ± 0.00b	3.66 ± 0.02ab	135 ± 1.41ab	123.50 ± 1.50a	262.50 ± 2.12a

Results are expressed as mean ± standard error. Values with different letters in the same column (a–d) are significantly different (P ≤ 0.05) from each other.

The dry matter values of the tomato juice after plasma processing were in the range of 3.44–3.69% (Table 2). The dry matter of samples treated with atmospheric cold plasma for 120 and 300 seconds was not statistically different from that of the non-treated sample (control). As reported by other authors, possibly an effect of the fan^46^ caused about 7% incensement in dry matter content just after the processing in comparison to the control samples. It was also observed by Wang *et al*.^47^.

Carotenoids are often present in fruits and vegetables and are essential biological compounds. Many research works emphasize, that carotenoids can serve as free-radical scavengers, anti-aging compounds, prevent aging, tissue damage, and heart disease^48^. Among them, lycopene has drawn a considerable interest as epidemiological studies revealed correlation between increased lycopene-rich foods intake (for instance products based on tomatoes) and lower risk of cancer^49^. In the pasteurized juice, substantially higher total carotenoid and lycopene content was recorded compared to the control sample. Under the influence of temperature, the content of lycopene increases and the compound is transformed into a form in which it is much better absorbed from the gastrointestinal tract into the body^4,50^.

Lycopene dominates among the carotenoids found in tomatoes. It was found that the content of this compound in was as high as 5 mg/100 g intensely red tomatoes and 0.5 mg/100 g in the yellow variety^51,52^. As a result of the action of cold plasma, there was a slight increase in the total carotenoid and lycopene content in the tomato juice (Table 2). The juice treated for 120 s with low temperature plasma was characterized by the maximum content of these compounds. The increase in the total carotenoid and lycopene content was 13% and 11%, respectively, relative to the control. The increase in the contents of carotenoids and lycopene in tomatoes as a result of pulsed electric field treatment was reported by González-Casado *et al*.^36^. It can result in an increase in the extractability from the tomato tissue matrix.

Vitamin C has an effective protective activity in coronary disease and has the ability to destroy lipid peroxides and neutralize the harmful effects of free radicals arising both during food preparation and as a result of metabolic processes in the organism. A deficiency of vitamin C in the diet increases the susceptibility of tissues to free radicals, both extrinsic and those that arise in the body as a result of increased oxidative processes.

The content of vitamin C in the control sample of tomato juice was 274.0 mg/100 g d.w. Decontamination with the use of cold plasma resulted in only small losses of vitamin C (maximum 5% at the 300-second processing) and these were not statistically significant differences compared to the control (Table 2). The reduction in the content of this compound may be related to the oxidative effect of the plasma and the possibility of degradation of vitamin C through UV radiation generated during the plasma treatment^47^.

The research results clearly show that the tested technology can be used for production of new natural food products characterised by a longer shelf life than one-day-old juices. Since sensory analysis is an integral part of the process of development of new food products, it is necessary to carry out sensory tests at the laboratory and consumer levels^53–59^. Therefore, further research will address the question whether the cold plasma technology used for production of tomato juice will yield a product with required sensory quality and whether the product will be accepted by consumers. Tests using 9 point hedonic scale, using a trained panel and employing combination tests with biometric measurements^55^ will be carried.

## Conclusions

The time of exposure was the main factor influencing the tomato juice treatment efficacy with low temperature plasma generated from air as a substrate gas. 5 minutes of plasma treatment enabled 3.45-log CFU/ml, 3.55-log CFU/ml and 3.32-log CFU/ml reductions just after the treatment for the total aerobic mesophilic bacteria colonies, yeast and molds, respectively. However, statistically significant reduction was obtained already after 30 s of the plasma treatment for the total viable count and after 60 s for the yeast and mold count.

The reduction effect was not only prolonged but also increased during the storage time at 4 °C following the plasma treatment. On the 3 day of storage, the number of colonies was reduced by 5.99 CFU/ml after 5 minutes of plasma treatment what was probably caused by oxidative species and products of their further reactions in juice after plasma treatment.

Dry matter and pH of samples treated with atmospheric cold plasma were not affected by plasma treatment. Slight increase in the total carotenoid (13%) and lycopene (11%) content was observed. Maximum 5% loss of vitamin C after 5 minutes treatment was measured.

Low temperature atmospheric pressure plasma generated in GAD reactor can be a potential tool for extending the shelf-life of tomato juice with preserving of selected bioactive compounds.
